# Immunomodulatory and immunosuppressive drug protocols in the treatment of canine primary immune thrombocytopenia, a scoping review

**DOI:** 10.1186/s13028-021-00620-z

**Published:** 2021-12-27

**Authors:** Peter Spanner Kristiansen, Lise Nikolic Nielsen

**Affiliations:** 1 Anicura Odense Dyrehospital, Rugaardsvej 105, 5000 Odense, Denmark; 2 Department of Clinical Veterinary Sciences, Faculty of Health and Medical Sciences, University of Copenhagen, Dyrlaegevej 16, 1870 Frederiksberg C, Denmark

**Keywords:** Dog, Glucocorticoids, Human immunoglobulins, Platelets, Therapy

## Abstract

Primary immune thrombocytopenia (ITP) is a cause of severe thrombocytopenia in dogs. Immunosuppressive corticosteroid drugs are frequently used in the management of ITP, but treatment failure may occur. Immunomodulatory and non-corticosteroid immunosuppressive drugs might improve outcomes from therapy either alone or in combination with corticosteroids. The objectives of this scoping review were (1) to evaluate the current evidence relating to immunomodulatory and immunosuppressive drug protocols in the treatment of canine ITP, and (2) to answer the clinical question, whether or not therapy with immunomodulatory or non-corticosteroid immunosuppressive drugs alone or in combination with corticosteroids could improve outcome, compared to therapy with corticosteroids alone. A literature search was performed in the electronic databases of Agricola, CAB Abstracts, Embase, Medline and Web of Science for publications in November 2019 and again February 1, 2021. Selection criteria were relatively strict and included peer-reviewed research papers reporting outcome measures from immunomodulatory and immunosuppressive drug protocols in the treatment of canine ITP with a pre-therapeutic mean or median platelet count < 50,000/µL as a strict criterion for inclusion. Studies were evaluated if they had an appropriate diagnostic work up to exclude underlying conditions. Outcome measures and adverse events were compared between drug protocols both within studies and between studies. The search identified 456 studies, with six studies being eligible for inclusion. The studies were mostly case series while two were randomized controlled trials. Level of evidence varied with an overall uncertain subject enrollment, small groups, inadequate description and variable use of drug protocols or outcome measures. For outcomes such as platelet recovery time and duration of hospitalization, an improvement was observed using adjunctive therapy (human intravenous immunoglobulin) compared to therapy with corticosteroids alone. For outcomes of complete platelet recovery time, survival (6-month), mortality and relapse, no improvement was observed using adjunctive drugs compared to corticosteroids alone. Specifically, therapy with mycophenolate mofetil alone and adjunctive azathioprine were associated with more severe adverse events compared to other drug protocols. Evidence relating to immunomodulatory and immunosuppressive drug protocols in the treatment of canine ITP was of variable quality. Future larger case-controlled trials are required for determination of optimal treatment protocols in canine ITP.

**Supplementary Information:**

The online version contains supplementary material available at 10.1186/s13028-021-00620-z.

## Background

Primary immune thrombocytopenia (ITP) is a common cause of severe thrombocytopenia in the canine population [1, 2]. ITP is a diagnosis of exclusion and requires absence of non-immunologic causes of platelet consumption, platelet sequestration and decreased platelet production [3, 4], as well as immunologic causes of thrombocytopenia secondary to underlying neoplastic, infective, inflammatory diseases and medications (secondary immune thrombocytopenia (sITP)) [4, 5]. ITP is recognized as a complex and heterogeneous disease that occurs from a combination of humoral and cell-mediated destruction of circulatory platelets and in rare cases megakaryocytes in the bone marrow [6, 7]. Life-threatening bleeding may occur in patients with severe thrombocytopenia, in particular when the platelet counts decrease below 30,000/µL or below 50,000/µL depending on the study [7–11].

Immunomodulatory and immunosuppressive drugs form the cornerstone of therapy for ITP [12]. While immunomodulatory drugs have selective actions in the adaptive immune system mediated by the regulatory subsets of the CD4^+^ T lymphocytes, immunosuppressive drugs like corticosteroids affect several parts of the immune response affecting both the innate and adaptive immune system resulting in a ‘blanket immunosuppression’ with potential beneficial but also deleterious effects [13]. Corticosteroids have historically been used as first-line therapy in ITP, but their efficiency have never been subjected to rigorous evaluation by randomized blinded placebo-controlled trials [12]. Treatment failure may be related to underlying drug resistance or adverse events related to high dosage therapy [12, 14]. Adjunctive drugs may have complementary immunologic effects. When used in combination with corticosteroids, they may improve outcomes and decrease severity of corticosteroid-related adverse events [13, 15]. Additionally, treatment with non-corticosteroid immunomodulatory or immunosuppressive drugs could be beneficial as monotherapy. This area has been subjected to research during the last two decades and a variety of immunomodulatory and immunosuppressive drug protocols have been investigated in an attempt to improve different outcome parameters [9, 11, 16–22]. To date, no studies have evaluated the quality of this evidence and no consensus recommendations is available relating to the management of canine ITP. The primary objective of this scoping review was to evaluate the current evidence relating to immunomodulatory and immunosuppressive drug protocols in the treatment of canine ITP. The secondary objective was to answer the clinical question whether or not therapy with immunomodulatory or non- corticosteroid immunosuppressive drugs alone or in combination with corticosteroids could improve outcome, compared to therapy with corticosteroids alone in canine ITP.

### Search strategy

A more in-depth search strategy can be found in Additional file 1.

### Protocol and registration

A review protocol was drafted using the checklist and explanation of the PRISMA Extension for Scoping Reviews (PRISMA-ScR) [23]. Published primary studies concerning immunomodulatory and immunosuppressive treatment of canine ITP are heterogeneous groups in relation to design, methods, materials, and outcome reporting, and therefore the scoping review format was selected. However, strict eligibility criteria for study selection were still used, as ITP is a diagnosis of exclusion and must be distinguished from other causes of thrombocytopenia [23].

Eligibility criteria included peer-reviewed research reports including randomized control trials (RCT), controlled clinical trials, cohort studies, case–control studies and case series reporting original data from dogs with ITP treated with protocols consisting of (1) corticosteroids alone, or (2) immunomodulatory or non-corticosteroid immunosuppressive drugs alone, or (3) immunomodulatory and/or non-corticosteroid immunosuppressive drugs in combination with corticosteroids reporting outcome measures were included. Studies reporting treatment groups with median or mean platelet count below 50,000/µL by an automated platelet count, which were verified by estimation on a stained blood smear were included. Studies excluding other causes of thrombocytopenia and underlying diseases in the diagnostic workup of ITP were selected. The outcome measures investigated were platelet recovery time, duration of hospitalization, complete platelet recovery time, survival to discharge, survival after discharge and relapse. These were selected, as they are commonly reported objective markers of short and long-term treatment efficiency in studies of canine ITP. In addition, adverse events related to treatment were included, as this parameter affects patient morbidity and mortality. The outcome measures had to be stated according to the drug protocol used with description of drug names and dosage range.

### Information sources

Studies were identified by searching in November 2019 and again February 1, 2021 in the electronic databases, Agricola (1970 to present), CAB Abstracts Archieve (1910 to present), Embase (1974 to present), and Medline (1946 to present) via Ovid^1^ and Web of Science^2^ (1970 to present).

### Search

The search strategy used in the search engines using Ovid were: (dog? OR canine) AND (immune OR immune-mediated OR immunity OR autoimmune) AND (IMT OR ITP OR IMTP OR thrombocytopenia OR thrombocytopenic purpura) AND (treatment? OR treated OR treat OR treating OR therapy OR therapies OR therapeutic? OR immunosupp* OR ciclosporin OR cyclosporine OR azathioprine OR prednisone OR prednisolone OR dexamethasone OR vincristine OR mycophenolate OR cyclophosphamide OR IVIG OR immunoglobulin OR globulin OR danazol OR leflunomide). The wildcard symbol ‘?’ substitutes for one character or none and the truncation symbol ‘*’ substitutes for strings of zero or more characters. In Web of science, the search terms were identical except for the wildcard symbol ‘?’ which were changed to ‘$’. Duplicates were removed and an abstract present were selected as limits using Ovid. The studies identified in Ovid and Web of Science were transferred to the electronic reference manager program Mendelay^3^ and processed to remove duplicates.

### Critical appraisal of individual sources of evidence

Level of evidence and methodological quality were assessed using the Scottish Intercollegiate Guidelines Network Grading System (SIGN Grading System 1999–2012) and critical appraisal checklists for RCT, controlled clinical trials, cohort studies and case–control studies (Table 1). SIGN checklists were selected according to the study design with aid of the SIGN algorithm for classifying study designs for questions of effectiveness. Eligible studies were graded by LOE on a scale of 1–4 according to the pyramid of evidence with a sub-classification in level 1 and 2. According to the critical appraisal checklists, RCTs and controlled clinical trial*s* were graded to be of *high, acceptable or low* methodological quality by evaluating the risk of bias. (1) A *high* quality was graded when the study had a very low risk of bias. (2) An *acceptable* quality was graded when the study had a low risk of bias, and (3) a *low* quality was graded when the study had a high risk of bias. Cohort studies and case–control studies were graded to be of *high*, *acceptable* or *low* methodological quality by evaluating the risk of bias or confounding factors, and the evidence of a relationship between treatment and outcome. The methodological quality of case series, case reports or expert opinion was not evaluated following the SIGN guidelines. The methodological quality of studies was additionally evaluated by the following two measures, size of treatment groups, and quality of subject enrollment. The strength of treatment group sizes was defined as *good*, *moderate*, *small*, or *very small* according to criteria used by previous veterinary systematic reviews [24–26]. In short, > 50 animals per group were categorized as *good*, 20–50 animals per group were categorized as *moderate*, 10–19 animals per group were categorized as *small*, and < 10 animals per group were categorized as *very small*. The quality of subject enrollment was graded as *strongly supportive, supportive*, or *uncertain* for building evidence for a diagnosis of canine ITP, according to diagnostic criteria proposed by two veterinary reviews [3, 4]. Diagnostic criteria were categorized into three groups. Each group was evaluated to see whether the criteria were fulfilled in all of the enrolled study participants or not (Table 2). Studies not specifying a number of animals subjected to a particular test were graded with an uncertain subject enrollment quality.

**Table 1:** The Scottish Intercollegiate Guidelines Network (SIGN) grading system 1999–2012

Level of evidence	Study design and methodological quality
Level 1^++^	High quality meta-analyses, systematic reviews of RCTs, or RCTs with a very low risk of bias
Level 1^+^	Well-conducted meta-analyses, systematic reviews, or RCTs with a low risk of bias
Level 1^−^	Meta-analyses, systematic reviews, or RCTs with a high risk of bias
Level 2^++^	High quality systematic reviews of case control or cohort studies High quality case control or cohort studies with a very low risk of confounding or bias and a high probability that the relationship is causal
Level 2^+^	Well-conducted case control or cohort studies with a low risk of confounding or bias and a moderate probability that the relationship is causal
Level 2^−^	Case control or cohort studies with a high risk of confounding or bias and a significant risk that the relationship is not causal
Level 3	Non-analytic studies, e.g. case reports, case series
Level 4	Expert opinion

Level of evidence by study design and methodological quality of interventional studies using the SIGN system. RCT, randomized controlled trials

**Table 2:** Grading of study subject enrollment

Diagnostic criteria	Grade			
	Strongly supportive	Supportive	Uncertain	
1) Initial verified automated platelet count < 50,000/µL in all animals	Yes	Yes	Yes	No
2) Exclusion of underlying diseases by hematologic and biochemical blood samples, urinalysis, coagulation panel testing, serology and/or PCR for infectious disease and diagnostic imaging of the thorax and abdomen in all animals	Yes	Yes	No	N/A
3) Detection of platelet autoantibodies and/or exclusion of underlying disease by bone marrow sampling in all animals	Yes	No	N/A	N/A

Quality of subject enrollment in studies graded as strongly supportive, supportive, or uncertain for building evidence for a diagnosis of canine primary Immune thrombocytopenia. The table was based on diagnostic criteria suggested by LeVine and Brooks [3], Heseltine and Carr [4] N/A not applicable, PCR polymerase chain reaction

### Synthesis of results

Aiming to answer the primary objective, a summary of the eligible studies LOE and methodological quality was evaluated. Study characteristics such as study design, drug protocol description, use of drug protocols and outcome measures were evaluated as well and with the aid of the PRISMA SIGN checklist (Additional file 2). Observations of the adverse events related to treatment protocols were graded on a scale from 1 to 5 using common terminology criteria for adverse events (VCOG‐CTCAE v2) following investigational therapy by the veterinary cooperative oncology group [27].

Aiming to answer the secondary objective and clinical question, reported outcome measures (platelet recovery time, duration of hospitalization, survival to discharge, survival after discharge, relapse rate) and adverse events from therapy were compared between drug protocols consisting of (1) corticosteroids alone, (2) immunomodulatory or non-corticosteroid immunosuppressive drugs alone, or (3) immunomodulatory and/or non-corticosteroid immunosuppressive drugs in combination with corticosteroids. An improvement in outcome was defined, when a significant difference in outcome measures between treatment and comparator was detected and if the treatment was superior to the comparator. No improvement in outcome was defined, when no significant difference in outcome measures between treatment and comparator was detected. No discrimination was made between difference in characteristics of study designs, study populations, severity of ITP, drug protocols (formulation, dosage range, frequency, time of administration) and extent of concomitant treatment in the analysis of outcomes.

## Review

### Selection of sources of evidence

**Fig. 1:**
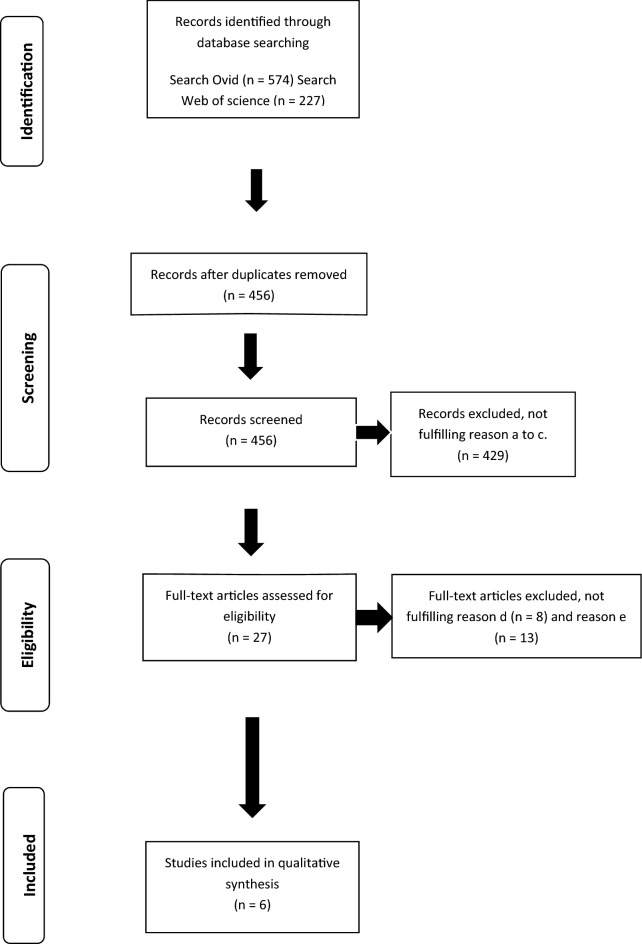
Flow diagram of study selection according to Preferred Reporting Items for Systematic Reviews and Meta-analyses (PRISMA). Study inclusion criteria: (a) peer-reviewed research reports; (b) original data reported in the research paper; (c) use of immunomodulatory and immunosuppressive drug protocols in the management of canine ITP; (d) and reporting outcomes and adverse events according to the used protocol with drug name and dosage range; (e) groups of dogs with ITP with a mean or median platelet count below 50,000/µL with exclusion of other causes of thrombocytopenia using history, physical examination, blood samples, coagulation panel testing, serology and/or polymerase chain reaction and diagnostic imaging

A total of 574 records were identified by the literature search strategy but only six studies fulfilled the criteria for inclusion in the qualitative synthesis (Fig. 1). The six studies reported outcomes from immunomodulatory and immunosuppressive drug protocols in the treatment of canine ITP.

### Characteristics of sources of evidence

Of the six eligible studies, two studies were RCT [18, 28], one study was a retrospective case series with a nested cohort [8], two studies were prospective case series [10, 17], and one study was a retrospective case–control study [29], but with a case series design for the therapeutic intervention (Table 3). One RCT was categorized with a multicenter and blinded design [18]. The other RCT was categorized with a double-blinded and placebo-controlled design [28]. All participants in the studies were client-owned dogs with a diagnosis of ITP. Dogs with a concurrent diagnosis of osteoarthritis were enrolled in one study [17]. The investigated study groups had an initial platelet count median of 1000/µL to a mean of 10,400/µL.

**Table 3:** Summary of eligible studies reporting outcomes from treatment with immunomodulatory and immunosuppressive drug protocols in canine primary ITP

Study	Level of evidence, study design, and methodological quality	Study population (range)	Intervention and comparator	Outcome and adverse events (range)	Statistical comparison (*P* value or [95% Cl])
Kohn et al. [7]	LOE 3 Prospective case series Very small number of dogs in each group Uncertain enrollment	15 client-owned dogs with a diagnosis of ITP with a mean initial platelet count of 10,400/µL (0–74,000/µL) 13 dogs reached the outcome	Treatment during beginning of disease: Tx_1_: Prednisolone 1–1.5 mg/kg BID Tx_2_: Prednisolone 1–1.5 mg/kg BID + Aza 1,5-2 mg/kg SID Tx_3_; Prednisolone 1–1.5 mg/kg BID + Aza 1.5-2 mg/kg SID (4–7 days after Vinc Inj) + Vinc 0.5 mg/m^2^ once or possibly after 1 week	Platelet recovery > 50.000/µL: Tx_1_: 1, 2, 3, 3, 4, 5 and 9 days Tx_2_: 3, 3 and 11 days Tx_3_: 2, 3 and 5 days Platelet recovery > 150,000/µL: Tx_1_: 3, 5, 6, 6, 6, 11 and 13 days Tx_2_: 6, 10 and 22 days Tx_3_: 4, 5 and 13 days Adverse events: Tx_2_ and Tx_3_: Grade 5 death	No statistical evaluation of outcome
Balog et al. [14]	LOE 1^+^ (Randomized), multicenter, blinded, clinical trial Small number of dogs in each group Uncertain enrollment	20 client-owned dogs with a diagnosis of severe primary ITPwith a median initial platelet count of 1000/µL (0–16,000/µL)	Treatment within 24 h of initial presentation to day 7: Tx_1_: Prednisone 1.5-2 mg/kg BID or Dex 0.2–0.3 mg/kg SID + hIVIG 0.5 g/kg once C: Prednisone 1.5-2 mg/kg BID or Dex 0.2–0.3 mg/kg SID + Vinc 0.02 mg/kg once Adjunctive treatment after day 7 if no platelet recovery occurred: Alternative drug (VINC or hIVIG) + Aza 2 mg/kg SID	Platelet recovery ≥ 40,000/µL: Tx_1_: median 2.5 days (0–10 days) C: median 2.5 days (1–4 days) Duration of hospitalization^a^: Tx_1_: median 5 days (1.5–10 days) C: median 4 days (3–5 days) Survival to discharge: Tx_1_: 70% C: 100% Survival 6-month: Tx_1_: 22% C: 70% Survival 1-year: Tx_1_: 22% C: 60% Adverse events: Tx_1_ and C: Grade 1 mild	No significant difference (*P* < 0.05) in platelet recovery time (*P* = 0.51), duration of hospitalization (*P* = 0.29), survival to discharge (*P* = .21), survival 6-months (P = 0.17) and survival 1-year (*P* = 0.07) between groups
Bianco et al. [26]	LOE 1^++^ Randomized, double-blinded, placebo-controlled, clinical trial Very small number of dogs in each group Strongly supportive enrollment	18 client-owned dogs with a presumptive diagnosis of primary ITP with a median initial platelet count of 2000/µL (1000–18,000/µL)	Treatment within 24 h of initial presentation to day 7: Tx_1_: Prednisone 1.5 mg/kg BID + hIVIG 0.5 g/kg once C: Prednisone 1.5 mg/kg BID + Placebo (0.9% NaCl) Adjunctive treatment on day 7 if no platelet recovery occurred or to decrease dosage of prednisone: Alternative drug (Placebo or hIVIG) + Aza 2 mg/kg SID, Vinc 0.02 mg/kg once and Cyclo 5 mg/kg BID in different combinations	Platelet recovery > 40,000/µL: Tx_1_: median 3,5 days (2–7 days) mean 3.7 ± 1.3 days SD C: median 7.5 days (3–12) mean 7.8 ± 3.9 days SD Duration of hospitalization^b^: Tx_1_: median 4 days (2–8) mean 4.2 ± 0.4 days SD C: median 8 days (4–12) mean 8.3 ± 0.6 days SD Platelet recovery > 160,000/µL: Tx_1_: median 8 days (3–19) C: median 13 days (5–32) Survival to discharge: Tx_1_: 100% C: not described Survival 6-month: Tx_1_:100% C:78% Relapse during 6-month^c^: Tx_1_: 11% C: 11% Adverse events: Tx_1_: Grade 1 mild C: Grade 1 mild	Significant difference (*P* < 0.05) in platelet recovery time > 40,000/µl (*P* = 0.018) and duration of hospitalization (*P* = 0.027) between groups No significant difference in platelet recovery > 160,000/µl (*P* = 0.093) or survival 6-months (*P* = 0.79) between the two initial groups and between all dogs that did not receive hIVIG and those that did at some point during the 6-month period (*P* = 0.53)
Putsche and Kohn [5]	LOE 3 Retrospective case series with a nested cohort study Small to very small number of dogs in each group Uncertain enrollment	30 client-owned dogs with a diagnosis of primary ITP with a median initial platelet count of 8000/µL (0–111,000/µL)	Treatment during disease: Tx_1_: Prednisolone 1–1.5 mg/kg BID Tx_2_: Prednisolone 1–1.5 mg/kg BID + Vinc 0.02 mg/kg (Fq not described) Tx_3_: Prednisolone 1–1.5 mg/kg BID + Aza 1.5-2 mg/kg SID Tx_4_: Prednisolone 1–1.5 mg/kg BID + Cyclo 5 mg/kg SID C: Tx_2_ + Tx_3_ + Tx_4_ Adjunctive treatment at beginning of treatment or 5–43 days later, if no platelet recovery occurred or after a relapse	Platelet recovery ≥ 50,000/µL: Tx_1_: median 5 days (4–11 days) mean 6 ± 2.2 days SD Tx_2_: median 4 days (2–7 days) mean 4 ± 2 days SD Tx_3_: 4, 7 and 12 days Tx_4_: 5 and 9 days Adverse events: Tx_3_: Grade 5 death	No significant difference (*P* < 0.05) in relapse (*P* = 0.676) and mortality (*P* = 0.367) between Tx_1_ and C during 1684 days. Relapse: 26% [9%–51%] and Mortality: 10% [2%–27%]
Huang et al. [27]	LOE 3 Retrospective (case- control study) Small to very small number of dogs in each group Uncertain enrollment	48 client-owned dogs with a diagnosis of presumptive primary ITP, some had recent vaccination, with a median initial platelet count of 1000/µL (0–39,500/µL)	Treatment during hospitalization: Tx_1_: Prednisone 1-4 mg/kg/day or Dex 0.04–0.5 mg/kg/day + Aza 2 mg/kg/day Tx_2_: Prednisone 1-4 mg/kg/day or Dex 0.04–0.5 mg/kg/day + Vinc 0.02 mg/kg once Tx_3_: Prednisone 1-4 mg/kg/day or Dex 0.04–0.5 mg/kg/day + hIVIG 0.35–0.81 g/kg once	Platelet recovery = 40,000/µL: Tx_1_: median 6 days (4–12) Tx_2_: median 4 days (2–10) Tx_3_: median 5 days (2–10) Platelet recovery to reach reference range: Tx_1_: median 15 days (12–21) Tx_2_: median 10 days (3–42) Tx_3_: median 12 days (2–13) Survival to discharge: Tx_1_: 70% Tx_2_: 90% Tx_3_: 83% No evaluation of adverse events	No statistical evaluation of outcome
Yau and Bianco [13]	LOE 3 Prospective case series Very small number of dogs in each group Uncertain enrollment	5 client-owned dogs with a diagnosis of presumptive ITP, chronically treated NSAIDs for osteoarthritis with a median initial platelet count of 3000/µL (1000–14,000/µL)	Treatment during beginning of disease: Tx_1_: Mycophenolate mofetil 7.1–14.4 mg/kg BID, median 8.5 mg/kg	Platelet recovery > 50,000/µL: Tx_1_: median 3 days (2–6 days) Duration of hospitalization: Tx_1_: median 3 days (2-7 days) Platelet recovery ≥ 170,000/µL: Tx_1_: median 9 days (5–16 days) Survival to discharge: Tx_1_: 100% Adverse events: Tx_1_: Grade 2 moderate	No statistical evaluation of outcome

Aza, azathioprine; BID, twice a day; C, comparator; (case–control study), investigating association between recent vaccination and ITP but case series for the therapeutic intervention; Cl, confidence interval [lower limit, upper limit]; Cyclo, cyclosporine; Dex, dexamethasone; Fq, frequency; hIVIG, human intravenous immunoglobulin; NSAIDs, non-steroidal anti-inflammatory drugs; Inj, injection; (randomized), study claimed to be randomized but procedure not described; LOE, level of evidence; SD, standard deviation; SID, once a day; Tx_1-4_, Immunomodulatory and/or immunosuppressive treatment; Vinc, Vincristine

^a^Defined: initial presentation to discharge when platelet counts ≥ 40,000/µL

^b^Defined: initial presentation to discharge when clinical stable and platelet counts > 40,000/µL

^c^Defined: a platelet count decrease of 50% compared to previous count or any count of < 40,000/µL after initial response

Five studies provided description of dosage, range and frequency of all immunomodulatory and immunosuppressive drugs used for therapy [17, 18, 28–30]. The remaining study provided description of dosage and range of all drugs, but frequency of one immunosuppressive drug was missing [8]. Information regarding duration of therapy for all drugs was not clearly described in any of the six studies. Description of how drugs were tapered during disease remission was provided in three studies, but this information was not provided for all the drugs administered [8, 17, 28]. Drug formulation, dosage range and frequency varied across studies and included prednisolone/prednisone 2–4 mg/kg/day, dexamethasone 0.04–0.5 mg/kg/day, mycophenolate mofetil 7.1–14.4 mg/kg/day, azathioprine 1.5–2 mg/kg/day, vincristine 0.02 mg/kg once or 0.5 mg/m^2^ once, human intravenous immunoglobulin 0.35–0.81 g/kg once, and cyclosporine 5–10 mg/kg/day. Of the drug protocols investigated, treatment with glucocorticoids alone were reported in three studies [8, 10, 17, 28], and treatment with mycophenolate mofetil alone was reported in one study [17]. Treatment with corticosteroids and one adjunctive drug (azathioprine, vincristine, human intravenous immunoglobulin or cyclosporine) were reported in five studies [8, 10, 18, 28, 29] and at least two treatment groups receiving different adjunctive drugs were reported in three studies [8, 18, 29]. Treatment with corticosteroids and two adjunctive drugs (vincristine and azathioprine) was reported in one study [10]. The four most commonly used drug protocols were corticosteroids alone (total of 35 dogs), corticosteroids and adjunctive vincristine (total of 26 dogs), corticosteroids and adjunctive human intravenous immunoglobulin (total of 25 dogs), and corticosteroids and adjunctive azathioprine (total of 16 dogs). Corticosteroids were administered in the initial treatment of ITP, but there was variation in the time and criteria of adjunctive drug administration between studies. Two studies reported use of adjunctive drugs, but did not specify time or criteria for administration clearly [10, 29].

Outcome measures of short-term treatment efficiency such as platelet recovery, complete platelet recovery, duration of hospitalization and survival to discharge were commonly used as endpoints of therapy (Table 3). Time of platelet recovery was expressed as mean, median or single values and the point of platelet recovery was variably defined hampering comparison across studies. The platelet recovery time was defined as the time to reach a platelet count ≥ 40,000/µL or ≥ 50,000/µL. The complete platelet recovery time was defined as the time to reach a platelet count > 150,000/µL [10], > 160,000/µL [28], or ≥ 170,000/µL [17]. One study reported a complete platelet recovery time until reaching the reference range, but specification of the reference range was missing [29]. Two studies defined duration of hospitalization from initial presentation to discharge, which occurred when dogs attained a platelet count ≥ 40,000/µL [18, 28]. Outcome measures of long-term treatment efficiency were infrequently reported and included survival (6-month, 1-year), and relapse (6-month). Only two studies reported survival 6-month and survival 1-year [18, 28], and just one study reported relapse 6-month [28]. Relapse was defined by a platelet count decrease of 50% compared to a previous count or any count of < 40,000/µL after initial response [28]. One study described an overall mortality and relapse during a 1684-day period from therapy with multiple drug protocols, but did not specify these outcomes according to each drug protocol [8]. This study defined relapse as a platelet count decrease < 150,000/µL after the platelet had already been within the reference range. Adverse events from treatment were reported in five studies [8, 10, 17, 18, 28], but were not described for all of the immunomodulatory and immunosuppressive drugs used in two of the studies [8, 10].

### Critical appraisal within sources of evidence

According to the study designs and methodological quality, the LOE was determined across studies. One RCT was categorized as LOE 1^++^ with a high methodological quality and a very low risk of bias [28]. The other RCT was categorized as LOE 1^+^ with an acceptable methodological quality and a low risk of bias [18]. The case series with a nested cohort study [8], the case–control study with case series design for the therapeutic intervention [29], and the two case series [10, 17] were categorized as LOE 3 (Table 3). The RCT categorized with an acceptable methodological quality claimed to be randomized, but the randomization method was not described clearly. In addition, clinicians and intensive care personnel were not blinded to treatment allocation, and blinding of owners was not stated by the authors. The RCT categorized with a high methodological quality had good randomization and allocation concealment. However, the intention to treat analysis was compromised, as two dogs were randomized and excluded prior to treatment. Differences in baseline variables were not significantly different between treatment groups in both studies. The dropout-rate of study participants was 10% in long term follow up in one treatment group treated with human intravenous immunoglobulins [18], while 0% drop-outs were reported in other treatment groups investigated in the two RCT studies [18, 28]. The statistical testing was appropriate for comparison of outcome measures between treatment groups in the RCT study with an acceptable methodological quality [18]. The other RCT study [28] used appropriate statistical tests for comparison of survival data between treatment groups, but did not specify methods for comparison of platelet recovery time and duration of hospitalization, which made it difficult to assess the statistical validity for these outcome measures. Post hoc and a priori power calculations were performed in both studies. Power calculations estimated that a study population of 20 dogs in each treatment group was needed to provide a power of 80% at 0.05 significance level to detect a 50% difference in median platelet recovery time between the treatment groups [28]. This result was in accordance with the other study, where a study population of 20 and 28 dogs in each treatment group were needed to provide a power of 80% at 0.05 significance level, to detect a difference in survival to discharge and 1-year survival between the treatment groups [18]. According to these power calculations, both studies were underpowered as no more than 9 to 10 dogs were included in each treatment group. Finally, pretreatment glucocorticoids were allowed up to 24–48 h before admission and other adjunctive drugs were allowed during treatment on or after day 7 in both studies, which was a potential source of confounding in the presented outcome measures.

Sizes of treatment groups were very small in three studies [10, 17, 28], very small to small in two studies [8, 29], and small in one study [18]. The quality of subject enrollment was categorized as uncertain for a diagnosis of ITP in five studies [8, 10, 17, 18, 29], and strongly supportive for a diagnosis of ITP in one study [28]. In the study categorized with the highest quality of subject enrollment [28] all of enrolled animals had an initial verified automated platelet count < 50,000/µL and were all subjected to diagnostic screening by blood analysis, urinalysis, coagulation panel testing, serology and/or PCR, diagnostic imaging and bone marrow sampling to exclude underlying diseases. In four studies, all of the study participants had an initial platelet count < 50,000/µL [17, 18, 28, 29], and in two studies a minority of dogs had an initial platelet count > 50,000/µL [8, 10]. Urinalysis was not performed in all study participants or not performed in three studies [17, 27, 29]. Coagulation panel testing was not performed in all study participants or the number of dogs subjected to testing, was not reported in two studies [10, 18]. Serology for infectious diseases known to be associated with thrombocytopenia, was not performed in all study participants or the number of dogs subjected to testing, was not reported in two studies [8, 10]. Platelet autoantibody testing was not performed in all study participants in four studies [17, 18, 28, 29] and finally, bone marrow sampling was only performed in all dogs in one study [28].

### Results of individual sources of evidence

The summary of outcomes from treatment with drug protocols within the individual studies can be seen in Table 3 (Grouping according to drug protocols please see Additional files 3, 4, 5). Three studies evaluated the difference in outcomes by comparative analysis between treatment protocols [8, 18, 28]. One RCT found a significant reduction in the platelet recovery time and duration of hospitalization with use of adjunctive human intravenous immunoglobulin compared to treatment with prednisone alone [28]. However, there was no significant difference in the complete platelet recovery time and survival 6-month between drug protocols. A nested cohort study found no significant difference in mortality and relapse rate during a 1684-day period with use of adjunctive azathioprine or vincristine, or cyclosporine in a pooled group compared to treatment with prednisolone alone [8]. The other RCT found no significant difference in platelet recovery time, duration of hospitalization, survival to discharge, survival 6-month, and survival 1-year with use of adjunctive human intravenous immunoglobulin and prednisone compared to treatment with adjunctive vincristine and prednisone [18].

Grade 1 mild adverse events were detected with use of prednisone alone in one study [28]. Grade 1 mild adverse events were observed with use of adjunctive vincristine and/or human intravenous immunoglobulin in combination with prednisone [18, 28]. Grade 2 moderate adverse events included diarrhea and decreased appetite with use of mycophenolate mofetil alone [17]. Grade 5 death adverse events due to severe pancreatitis and disseminated intravascular coagulation resulting in death were observed in dogs treated with adjunctive azathioprine [8, 10]. Adverse events from cyclosporine administration were not evaluated in any of the studies.

### Synthesis of results

For an overview of synthesis of results, please review Table 4. The majority of studies were case series with an LOE 3, and only two RCT were identified with an LOE 1^+^-LOE 1^++^ with a high to acceptable methodological quality due to a very low to low risk of bias. Five studies had an overall uncertain subject enrollment, while the remaining study [28] had a strongly supportive subject enrollment. Most studies had inadequate description of drug protocols, variable use of drug protocols and variable outcome measures. Risk of confounding and low statistical power were additional limitations in studies, making comparative analysis between drug protocols challenging.

**Table 4:** Synthesis of results of eligible studies reporting outcomes from immunomodulatory and immunosuppressive drug protocols in the treatment of canine primary ITP

**Study**	Bianco et al. [28]	Balog et al. [18]	Huang et al. [29]	Putsche and Kohn [8]	Kohn et al. [10]	Yau and Bianco [17]
Characteristics	Randomized, double-blinded, placebo-controlled, clinical trial	(Randomized), multicenter, blinded, clinical trial	Retrospective (case–control study)	Retrospective case series with a nested cohort study	Prospective case series	Prospective case series
Critical appraisal	LOE 1^++^ Very small number of dogs in each group Strongly supportive enrollment Low statistical power and risk of confounding	LOE 1^+^ Small number of dogs in each group Uncertain enrollment Low statistical power and risk of confounding	LOE 3 Small to very small number of dogs in each group Uncertain enrollment	LOE 3 Small to very small number of dogs in each group Uncertain enrollment	LOE 3 Very small number of dogs in each group Uncertain enrollment	LOE 3 Very small number of dogs in each group Uncertain enrollment
Results of individual sources	Tx_1_: CS + hIVIG C: CS Improvement^a^(Tx_1_): Platelet recovery Duration of hospitalization No improvement^b^: Complete platelet recovery Survival 6-month	Tx_1_: CS + hIVIG C: CS + Vinc No improvement^b^: Platelet recovery Duration of hospitalization Survival to discharge Survival 6-month Survival 1-year	N/A	Tx_1_: CS Tx_2_: CS + Vinc Tx_3_: CS + Aza Tx_4_: CS + Cyclo C: Tx_2_ + Tx_3_ + Tx_4_ No improvement^b^: Mortality 1684-days Relapse 1684-days	N/A	N/A

Summary of the study characteristics, the level of evidence and methodological quality by critical appraisal, and the results of comparative studies

Synthesis of results of eligible studies reporting outcomes from immunomodulatory and immunosuppressive drug protocols in the treatment of canine primary ITP. Summary of the study characteristics, the level of evidence and methodological quality by critical appraisal, and the results of comparative studies

Aza, azathioprine; C, comparator; Cyclo, cyclosporine; CS, Corticosteroids; (case–control study), investigating association between recent vaccination and ITP but case series for the therapeutic intervention; hIVIG, human intravenous immunoglobulin; (randomized), study claimed to be randomized but procedure not described; LOE, level of evidence; Tx_1-4_, Immunomodulatory and/or immunosuppressive treatment; Vinc, Vincristine

^a^Improvement in outcome was defined when a significant difference in outcome measures between treatment and comparator was detected and if the treatment was superior to the comparator

^b^No improvement in outcome was defined when no significant difference in outcome measures between treatment and comparator was detected

N/A, Non-applicable, as these studies did not provide statistical information between groups

Two studies reported outcomes with use of adjunctive immunomodulatory or non-corticosteroid immunosuppressive drugs in comparison to corticosteroids alone in the treatment of canine ITP [8, 28]. For outcomes of platelet recovery time and duration of hospitalization, an improvement was observed using adjunctive non-corticosteroid immunosuppressive drugs compared to corticosteroids alone. For outcomes of complete platelet recovery time, survival (6-month), mortality (1684-days) and relapse (1684-days), no improvement was observed using adjunctive immunomodulatory or non- corticosteroid immunosuppressive drugs compared to corticosteroids alone. Therapy with mycophenolate mofetil alone and adjunctive azathioprine was associated with grade 2 moderate and grade 5 death adverse events respectively, compared to grade 1 mild adverse events with therapy of other drug protocols.

## Summary of evidence

The primary objective of this scoping review was to evaluate the current evidence relating to immunomodulatory and immunosuppressive drug protocols in the treatment of canine ITP in a stringent manner. Numerous studies have reported use of drug protocols with corticosteroids, vincristine, azathioprine, human intravenous immunoglobulin, mycophenolate, cyclophosphamide and leflunomide in different combinations in the management of canine ITP [7–9, 11, 17–20, 28, 29, 31–36]. Unfortunately, most studies were excluded in this scoping review during the selection process due to lack of fulfilling one or more of the criteria for inclusion. Eight studies were excluded due to inadequate description of drug protocols, or outcomes and adverse events were not stated according to each drug protocol used. Thirteen studies were excluded due to a combination of failing the criteria’s of reporting a verified median or mean platelet count below 50,000/µL in the treatment groups, or lacking usage of coagulation panel testing, serology and/or polymerase chain reaction for infectious disease, or diagnostic imaging of the thorax and abdomen in the screening for underlying disease. Only six studies were included in the review and still the evidence was generally only of a variable quality. The majority of included studies were restricted by a combination of case series designs, uncertain subject enrollment, small sizes of treatment groups, poor drug protocol description, variable use of drug protocols and variable outcome measures.

### Size of treatment groups

Treatment group sizes were very small to small across the six studies. This could be related to a relatively low incidence of the disease across the canine population and difficulty in recruiting enough animals to these studies, despite at least one study being a multicenter study [18]. One epidemiologic survey identified that immune thrombocytopenia was relatively rare, with 48 dogs out of 987 cases documented with thrombocytopenia. Of these, approximately half of them were diagnosed with presumptive ITP [1]. In another retrospective study of 871 dogs with thrombocytopenia, 31 dogs were diagnosed with presumptive ITP [2]. Furthermore, as ITP is a diagnosis of exclusion, the comprehensive diagnostic strategy can be financially limiting for owners, leaving veterinarian no choice, but to make a presumptive diagnosis of ITP. This represents a further limitation to case selection. As a result of these findings, it would be a strong recommendation to increase treatment group sizes by using multicenter study designs, when planning future comparative trials evaluating the effect of drug protocols in therapy for canine ITP.

### Details of drug protocols and outcome measures

The six studies in this review failed to provide important information about the drug protocols administered, usually not providing a duration or time of drug administration, nor specifying protocols for tapering of drugs during disease remission. Many studies had retrospective designs and collected data over years, accordingly there was a risk of losing important information about the drug protocols used in therapy. There was considerable variation in the time and choice of immunomodulatory and immunosuppressive drugs during therapy. This could be attributable to the lack of consensus for treatment of dogs with primary and secondary immune thrombocytopenia and uncertainty to the definition of criteria (no platelet recovery, relapse and severe thrombocytopenia) for administration of adjunctive drugs. Another explanation could be the descriptive or retrospective designs of many studies, which made it impossible to use standardized treatment protocols. The effect of immunomodulatory and immunosuppressive drugs was investigated over a short period in most studies, commonly from admission and 2 to 4 weeks later. Outcome definitions and results from treatment had widely different forms among the studies investigated. Only three studies evaluated outcomes after hospital discharge or made comparative analysis, which made it difficult to access the efficacy of drug protocols and especially over a long-term period. Platelet recovery time was the most prevalent outcome measure, often defined by a cut-off platelet count, where the risk of spontaneous bleeding was low. However, platelet recovery time seems to have downsides as an outcome measure in ITP. Reaching a specific platelet count does not reliably predict the risk of clinically relevant bleeding [37]. Platelet dysfunction in addition to destruction of platelets is observed in ITP and overt bleedings are possible in dogs with similar platelet counts as dogs that do not bleed [7, 38]. Furthermore, it is undetermined whether a faster platelet recovery time is associated with a better long-term outcome in canine ITP. One study reported a slower platelet recovery time in a group of dogs experiencing relapse, compared to a group of dogs that did not relapse during a 1-year period, but these results have not been reproduced by others [21].

### Corticosteroids alone or with other immunosuppressive agents for ITP?

The secondary objective was to answer the clinical question whether or not therapy with immunomodulatory or non-corticosteroid immunosuppressive drugs alone or in combination with corticosteroids improves outcome compared to therapy with corticosteroids alone. This question could not be answered as only two included studies made comparisons between treatment with corticosteroids alone and other drug protocols. One RCT found a significant reduction in the short-term outcomes of platelet recovery time and duration of hospitalization with use of adjunctive human intravenous immunoglobulin, however no significant difference was detected in the outcomes of complete platelet recovery time and survival 6-month compared to corticosteroids alone [28]. The lack of improvement in the long-term outcomes of treatment with use of adjunctive drugs was supported by the nested cohort study. This study reported no significant difference in mortality and relapse during a 1684-day period, when using adjunctive azathioprine, or vincristine, or cyclosporine in drug protocols compared to corticosteroids alone [8]. However, small to very small sizes of treatment groups were adversely affecting the statistical power with the risk of a type 2 error in these studies. The nested cohort study had 12–17 dogs in each treatment group and the RCT had nine dogs in each treatment group. Power calculations were not performed in the nested cohort study, but if one were to transfer the power calculations from other studies, it would seem that the nested cohort study was underpowered. According to the power calculations made by both RCT, at least 20 dogs were needed in each treatment group to detect a significant difference in platelet recovery and survival to discharge, while at least 28 were needed in each treatment group to detect a significant difference in survival 1-year to provide adequate power [16, 18, 28]. Additional treatment was allowed or administered in both studies, which was a potential source of confounding that could invalidate study results. In the RCT, pretreatment with corticosteroids 24 h prior to admission were allowed and other adjunctive drugs were allowed after seven days of treatment. In the nested cohort study treatment with a corticosteroid, an antibiotic and vitamin K drugs were administered in 12 dogs for an unknown duration prior to admission. Furthermore, adjunctive treatment was administered in this study at the beginning of therapy, or 5–43 days later in dogs being refractory to treatment or experiencing relapse. This could have introduced bias as some dogs in the adjunctive drug group were more severe cases compared to the glucocorticoids alone group. Other studies excluded from this review have investigated the effect of adjunctive drugs in combination with corticosteroids compared to corticosteroids alone. Four studies did not observe a significant difference in survival to discharge [7, 9], complete platelet recovery time [11], and relapse [9, 21] using different adjunctive immunomodulatory or immunosuppressive drug combinations in therapy of canine presumptive ITP compared to corticosteroids alone [19]. One study reported significant reduction in platelet recovery time and duration of hospitalization with administration of adjunctive vincristine compared to corticosteroids alone [19]. However, these observations concerning the effects of adjunctive drugs were not documented by controlled trials or limited to very small numbers of cases. As mentioned previously, these excluded studies did not perform diagnostic screening to exclude underlying disease or did not provide adequate numerical information of the outcome according to each drug protocol used.

### Adverse events following immunomodulatory therapy

Grade 5 death adverse events were reported in two out of six dogs with adjunctive azathioprine treatment in two studies [8, 10]. Grade 2 moderate adverse events were reported in two out of five dogs with mycophenolate mofetil treatment alone in one study [17]. Occurrence and severity of adverse events can be dosage-dependent, however the dosages described for azathioprine (1.5–2 mg/kg/day) and mycophenolate mofetil (14.2–28.8 mg/kg/day) are considered standard dosages, when extrapolating information from reviews of immunomodulatory and immunosuppressive drugs used in the management of canine immune mediated disease [13, 14, 39, 40]. Similar observations are reported in the treatment of other canine immune-mediated diseases and these drugs should always be used with care in therapy of ITP [41, 42]. Nevertheless, larger prospective studies investigating the safety of mycophenolate mofetil and azathioprine are lacking and it is therefore difficult to draw firm conclusions in this review as treatment groups were very small and other drugs were administered simultaneously.

### Subject enrollment challenges in the studies

Five studies were categorized with an uncertain subject enrollment as other underlying causes of thrombocytopenia were not meticulously excluded in all study participants for a diagnosis of ITP. No consensus exists for building evidence for a diagnosis in canine ITP, and this increases the diversity of test selection between clinicians. Lack of standardized protocols for inclusion of study participants in especially retrospective studies was another factor limiting the overall enrollment quality. Two studies enrolled dogs with a platelet count > 50,000/µL as a platelet cutoff value of < 150,000/µL was used for inclusion. Although platelet counts overlap between ITP and sITP, the former usually is associated with platelet counts < 50,000/µL and in the majority of cases < 20,000/µL, which potentially could have increased the risk of a mixture of ITP and sITP in these two studies [43]. Most studies performed neither urinalysis nor coagulation panel testing in all study participants or at least, this was not reported. One study claimed to use coagulation panel testing as part of the inclusion criteria, but the number of tested dogs was not stated. Performing urinalysis and coagulation panel testing is important in the screening for underlying diseases as disseminated intravascular coagulation, nephropathies and urinary tract inflammation were identified in a considerable proportion of 871 dogs with thrombocytopenia in one study [1]. Serology for infectious disease known to be associated with thrombocytopenia was not performed in all study participants, or the number of dogs subjected to serological testing was not reported in two studies. Application of serological testing was often based on clinician preference in the studies and by estimation of local risk of exposure to infectious disease. This approach resulted in differences in the application of serological tests within study groups and between studies. Single titer serology was generally used, and only one study used convalescent titers 3 to 4 weeks apart. Diagnostic imaging of the thorax and abdomen was performed in all studies. Four studies did not use abdominal ultrasonography for detection of underlying neoplasia in all study participants. Instead, abdominal radiography was selected, which is inferior to ultrasonography for detection of abdominal pathology [44]. Only two out of the six studies used platelet autoantibody tests in order to have evidence of an immune-mediated process. Currently, platelet autoantibody tests are not widely available, and they cannot differentiate between cases of ITP and sITP, which limits their applicability in the initial screening [5, 43, 45, 46]. In five studies, bone marrow examinations were not performed in all study participants. It is considered that bone marrow examination should only be performed if there is a suspicion of underlying marrow disease, for example different types of pancytopenia, poor response to therapy, or in geriatric dogs, where the suspicion of underlying disease is high [3].

## Limitations of this review

This scoping review has limitations that need to be considered when interpreting the results. Evaluation of enrollment quality was based on diagnostic criteria proposed by two veterinary reviews for building supportive evidence for a diagnosis of canine ITP [3, 4]. As mentioned, there is no standardized diagnostic workup of these patients. The diagnostic workup of ITP is further complicated as there is neither reliable clinical nor laboratory parameters that allow accurate diagnosis. This made construction of a grading scheme for enrollment quality and setting diagnostic criteria for inclusion of studies difficult. The requirement of a manual verification of an automated platelet counts suggesting thrombocytopenia might have excluded studies erroneously [22, 31, 34]. While some studies would provide details with regards to a manual verification of an automated hematological analysis, other studies may have considered the manual evaluation as an intrinsic part of best practice and therefore did not provide this information separately leading us to exclude the study. Historic data of study participants were not included in the evaluation of the enrollment quality. Several studies reported recent exposure to drugs, vaccination and travel history, which increased the risk of sITP being inadvertently enrolled. However, these studies were already categorized with an uncertain enrollment and the analysis did not seem compromised. Difference in the drug protocols (formulation, dosage range, frequency, time of administration) and extent of concomitant treatment between studies were not evaluated in the analysis of outcomes, which could have affected the conclusions. Clinical outcomes and adverse events from immunomodulatory and immunosuppressive therapy were the primary focus in this review. Cost of treatment and requirements of transfusion are recognized as secondary outcome measures in canine ITP. These outcomes were excluded in the evaluation, as there are wide differences in the pricing and protocols for transfusion between hospitals. Differences in characteristics of study populations, severity of disease and presence of possible negative prognostic markers (elevated blood urea nitrogen, melena, and megakaryocytic aplasia) between studies were additionally not included in the analysis [6, 7, 34].

### Future treatment optimization in ITP

To obtain appropriate immunosuppression without adverse events can be dependent on the dosage of the immunomodulatory and immunosuppressive drug administered. Therapeutic drug monitoring and use of pharmacodynamic assays to measure immunosuppressive effects of drugs on the immune system is recognized as potential useful tools to optimize drug dosages in canine immune-mediated disease, but is currently investigated in only a few studies [47, 48]. Targeted immune-therapy, where one alters single immunological parameters without causing ‘blanket immunosuppression’ thereby potentially reducing adverse events, is another area for future research, which could be beneficial in management of ITP [12]. Humans with ITP may have inappropriately normal levels of thrombopoietin (TPO), which is the major regulator of platelet production [49]. Assays to measure canine TPO are not currently commercially available, and it is unknown whether dogs with ITP have similar problems with regulators of platelet production. However, novel therapeutic agents targeting the TPO receptors have yielded promising results in canine ITP refractory to conventional immunosuppressive therapy in one pilot study, but the area requires further research [30].

## Conclusion

When applying stringent inclusion and exclusion criteria to the patient population, there was little published evidence concerning immunomodulatory and immunosuppressive therapy in canine ITP. The evidence was generally of variable quality as the majority of studies were limited by case-series designs, uncertain subject enrollment, small sizes of study groups, inadequate drug protocol description, variable use of drugs protocols, and variable outcome measures. Most studies investigated effect of drug protocols over a short-term period and only three studies made comparative analysis between drug protocols. Compared to corticosteroids alone, adjunctive human intravenous immunoglobulin improved outcomes of platelet recovery time and duration of hospitalization, when used in initial treatment of canine ITP. Compared to corticosteroids alone, adjunctive immunomodulatory or non-corticosteroid immunosuppressive drugs did not improve outcomes of complete platelet recovery, survival (6-month), mortality (1684-days) and relapse (1684-days), when used in treatment of canine ITP. However, these two studies were limited by a combination of low statistical power, risk of confounding, a nested cohort design and uncertain enrollment, which made it difficult to draw firm conclusions. Therapy with mycophenolate mofetil alone and adjunctive azathioprine were associated with grade 2 moderate and grade 5 death adverse events, respectively. According to this observation, these drugs should be used with care for therapy of ITP and close monitoring is warranted. The findings made in this review, highlight several problems in the current evidence relation to immunomodulatory and immunosuppressive drug protocols in the treatment of canine ITP. There is a need for development of a standardized diagnostic work up of ITP, standardized drug administration and standardized outcome measures to evaluate therapy between studies. Larger prospective multicenter studies investigating drug protocols optimally over a long-term period are warranted to determine effective treatment protocols in canine ITP.

## Supplementary Information

**Additional file 1:** Complete search strategy.

**Additional file 2:** SIGN checklists for controlled trials (http://prisma-statement.org/prismastatement/Checklist.aspx=

**Additional file 3:** Outcomes from treatment with corticosteroids alone in canine primary ITP.

**Additional file 4:** Outcomes from treatment with immunomodulatory or non-corticosteroid immunosuppressive drugs alone in canine primary ITP.

**Additional file 5:** Outcomes from treatment with adjunctive immunomodulatory or non-corticosteroid immunosuppressive drugs in canine primary ITP.

## Data Availability

The list of references used and/or analyzed during the current study are available from the corresponding author on reasonable request.

## Abbreviations

ITP: Immune thrombocytopenia
LOE: Level of evidence
RCT: Randomized control trial
sITP: Secondary immune thrombocytopenia

## Footnotes

1 See: http://ovid.uk.ovid.com.

2 See: http://apps.webofknowledge.com.

3 See: http://mendelay.com.
