# Supplementary material for: Immunomodulatory and immunosuppressive drug protocols in the treatment of canine primary immune thrombocytopenia, a scoping review
Source: Acta Vet Scand. 2021 Dec 27;63:54. doi: 10.1186/s13028-021-00620-z (PMC8721564; doi:10.1186/s13028-021-00620-z)
Supplement: Supplementary file 1 — Additional file 1: Complete search strategy. [file 13028_2021_620_MOESM1_ESM.docx]

Additional file 1: complete search strategy

*Protocol and registration:*

This scoping review was developed as result of a postgraduate master thesis' project in the Internal Medicine specialization track of the Master of Companion Animal Sciences, University of Copenhagen. The secondary research question were formulated following the PICO format using an evidence-based veterinary medicine toolkit from the RCVS knowledge website.^A^ A review protocol was drafted using the checklist and explanation of the PRISMA Extension for Scoping Reviews (PRISMA-ScR) [23]. As the number of published primary studies concerning immunomodulatory and immunosuppressive treatment of canine ITP is a heterogeneous group in relation to design, methods, materials, and outcome reporting, the scoping review format was selected. However, strict eligibility criteria for study selection were used, as ITP is a diagnosis of exclusion and must be distinguished from other causes of thrombocytopenia due to therapeutic and prognostic differences between diseases.

*Eligibility Criteria:*

Peer-reviewed research reports including randomized control trials (RCT), controlled clinical trials, cohort studies, case-control studies and case series reporting original data from dogs with ITP treated with protocols consisting of (1) corticosteroids alone, or (2) immunomodulatory or non-corticosteroid immunosuppressive drugs alone, or (3) immunomodulatory and/or non-corticosteroid immunosuppressive drugs in combination with corticosteroids reporting outcome measures were included. Studies reporting treatment groups with median or mean platelet count below 50,000/µL by an automated platelet count, which were verified by estimation on a stained blood smear were included. Studies excluding other causes of thrombocytopenia and underlying diseases in the diagnostic workup of ITP through history, physical examination, hematologic and biochemical analysis of blood samples, coagulation panel testing, serology and/or Polymerase Chain Reaction (PCR) for infectious disease and diagnostic imaging of the thorax and abdomen were selected. The outcome measures investigated were platelet recovery time, duration of hospitalization, complete platelet recovery time, survival to discharge, survival after discharge and relapse. These were selected, as they are commonly reported objective markers of short and long-term treatment efficiency in studies of canine ITP. In addition, adverse events related to treatment were included, as this parameter affects patient morbidity and mortality. The outcome measures had to be stated according to the drug protocol used with description of drug names and dosage range.

*Information sources:*

Studies were identified by searching from November 2019 to February 1, 2021 in the electronic databases, Agricola (1970 to present), CAB Abstracts Archieve (1910 to present), Embase (1974 to present), and Medline (1946 to present) via Ovid^B^ and Web of Science^C^ (1970 to present). No restrictions were applied on publication date or language, but it was a requirement that the abstract was in English. Two reviewers (PSK and LNN) were involved in the search. The first reviewer (PSK) conducted the initial searches, and the second reviewer (LNN) was using the search string developed by the first reviewer for the confirmatory search.

*Search:*

The search strategy used in the search engines using Ovid were: (dog? OR canine) AND (immune OR immune-mediated OR immunity OR autoimmune) AND (IMT OR ITP OR IMTP OR thrombocytopenia OR thrombocytopenic purpura) AND (treatment? OR treated OR treat OR treating OR therapy OR therapies OR therapeutic? OR immunosupp* OR ciclosporin OR cyclosporine OR azathioprine OR prednisone OR prednisolone OR dexamethasone OR vincristine OR mycophenolate OR cyclophosphamide OR IVIG OR immunoglobulin OR globulin OR danazol OR leflunomide). The wildcard symbol ‘?’ substitutes for one character or none and the truncation symbol ‘*’ substitutes for strings of zero or more characters. In Web of science, the search terms were identical except for the wildcard symbol ‘?’ which were changed to ‘$’. Duplicates were removed and an abstract present were selected as limits using Ovid. The studies identified in Ovid and Web of Science were transferred to the electronic reference manager program Mendelay^D^ and processed to remove duplicates.

*Selection of sources of evidence:*

Titles and abstracts of the retrieved articles were reviewed manually by the reviewers. Relevant studies assessed by the authors to potentially fulfill the criteria of eligibility, were retrieved as full text studies. Studies in other languages than English were translated to English using Google Translate. Full text studies were assessed using the stated eligibility criteria for inclusion. When in doubt of a study’s eligibility, the study was discussed by both reviewers. Differences between the two authors were resolved by consensus before including or excluding the study. The reference lists from retrieved studies and from background literature were manually screened to identify studies missed by the initial electronic search.

*Data charting process:*

Data were extracted in tubular form independently by the both authors using a data extraction sheet developed for this review. The data extraction sheet developed and tested prior to use to ensure functionality by both authors using data extraction templates and data from the postgraduate master thesis' projects. The data collecting process were performed without blinding of publication titles, site, institution and author names from the reviewer.

*Data item:*

Information was collected according to the study characteristics of design, population, intervention, comparison and outcome. Data was collected from (1) the characteristics of the population of dogs enrolled (including history, group sizes and initial platelet counts). Information of the diagnostic workup performed was collected as well (including hematologic and biochemical blood sampling, urinalysis, coagulation panel testing, infectious disease serology and/or PCR, diagnostic imaging, platelet autoantibody testing and bone marrow sampling). (2) Data of the therapeutic intervention were collected (including drug formulation, dosage range, frequency, duration and time of administration). (3) Information of outcome measures (including platelet recovery time, duration of hospitalization, survival to discharge, survival after discharge and relapse) and adverse events from therapy was retrieved. In addition, application of statistical tests and results of comparative analysis were collected. Calculations of incomplete outcome data sets were not performed.

*Critical appraisal of individual sources of evidence:*

The level of evidence (LOE) and methodological quality of eligible studies included in this review were assessed by PSK and LNN. Doubt of a study’s level of evidence and quality, or disagreements between the two reviewers were resolved by consensus. Study designs such as randomized control trials (RCT), cohort studies, case-control studies and case series were included in the review to provide an overview of the existing evidence regarding the use of immunomodulatory and immunosuppressive drug protocols in treatment of canine ITP. Level of evidence and methodological quality were assessed using the Scottish Intercollegiate Guidelines Network Grading System (SIGN Grading System 1999-2012) and critical appraisal checklists for RCT, controlled clinical trials, cohort studies and case-control studies (Table 1). SIGN checklists were selected according to the study design with aid of the SIGN algorithm for classifying study designs for questions of effectiveness. Eligible studies were graded by LOE on a scale of 1-4 according to the pyramid of evidence with a sub-classification in level 1 and 2.

According to the critical appraisal checklists, RCTs and controlled clinical trial*s* were graded to be of *high, acceptable or low* methodological quality by evaluating the risk of bias. (1) A *high* quality was graded when the study had a very low risk of bias. (2) An *acceptable* quality was graded when the study had a low risk of bias, and (3) a *low* quality was graded when the study had a high risk of bias. Cohort studies and case-control studies were graded to be of *high*, *acceptable* or *low* methodological quality by evaluating the risk of bias or confounding factors, and the evidence of a relationship between treatment and outcome. The methodological quality of case series, case reports or expert opinion was not evaluated following the SIGN guidelines.

The methodological quality of studies was additionally evaluated by the following two measures, size of treatment groups, and quality of subject enrollment. The strength of treatment group sizes was defined as *good*, *moderate*, *small*, or *very small* according to criteria used by previous veterinary systematic reviews[24-26]. In short, >50 animals per group were categorized as *good*, 20-50 animals per group were categorized as *moderate*, 10-19 animals per group were categorized as *small*, and <10 animals per group were categorized as *very small*. The quality of subject enrollment was graded as *strongly supportive, supportive*, or *uncertain* for building evidence for a diagnosis of canine ITP, according to diagnostic criteria proposed by two veterinary reviews [3, 4]. Diagnostic criteria were categorized into three groups. Each group was evaluated to see whether the criteria were fulfilled in all of the enrolled study participants or not (Table 2). Studies not specifying a number of animals subjected to a particular test, were graded with an uncertain subject enrollment quality.

*Synthesis of results:*

Aiming to answer the primary clinical question, a summary of the eligible studies LOE and methodological quality was evaluated. Study characteristics such as study design, drug protocol description, use of drug protocols and outcome measures were evaluated as well and with the aid of the PRISMA SIGN checklist (additional file 1). Observations of the adverse events related to treatment protocols were graded on a scale from 1 to 5 using common terminology criteria for adverse events (VCOG‐CTCAE v2) following investigational therapy by the veterinary cooperative oncology group [27]. In brief, *Grade 1* was characterized by mild; asymptomatic or mild symptoms; clinical signs or diagnostic observations only; intervention not indicated. *Grade 2* was characterized by moderate; outpatient or non-invasive intervention indicated; moderate limitation of activities of daily living. *Grade 3* was characterized by severe or medically significant but not immediately life‐threatening; hospitalization or prolongation of hospitalization indicated; disabling; significantly limiting activities of daily living. *Grade 4* was characterized by life‐threatening consequences; urgent interventions indicated. *Grade 5* was characterized by death related to adverse events; can be defined as either euthanasia or natural death, according to the investigators’ discretion.

Aiming to answer the secondary clinical question, reported outcome measures (platelet recovery time, duration of hospitalization, survival to discharge, survival after discharge, relapse rate) and adverse events from therapy were compared between drug protocols consisting of (1) corticosteroids alone, (2) immunomodulatory or non-corticosteroid immunosuppressive drugs alone, or (3) immunomodulatory and/or non-corticosteroid immunosuppressive drugs in combination with corticosteroids. An improvement in outcome was defined, when a significant difference in outcome measures between treatment and comparator was detected and if the treatment was superior to the comparator. No improvement in outcome was defined, when no significant difference in outcome measures between treatment and comparator was detected. No discrimination was made between difference in characteristics of study designs, study populations, severity of ITP, drug protocols (formulation, dosage range, frequency, time of administration) and extent of concomitant treatment in the analysis of outcomes.

Footnote A: https://knowledge.rcvs.org.uk/document-library/ebvm-toolkit-1-asking-an-answerable-clinical-question/
