# Supplementary material for: Immunomodulatory and immunosuppressive drug protocols in the treatment of canine primary immune thrombocytopenia, a scoping review
Source: Acta Vet Scand. 2021 Dec 27;63:54. doi: 10.1186/s13028-021-00620-z (PMC8721564; doi:10.1186/s13028-021-00620-z)
Supplement: Supplementary file 3 — Additional file 3: Outcomes from treatment with corticosteroids alone in canine primary ITP. [file 13028_2021_620_MOESM3_ESM.docx]

Additional file 3. Outcomes from treatment with corticosteroids alone in canine primary ITP.

| Study and protocol | Number of dogs | Platelet recovery time | | Duration of hospitalization | Survival to discharge | Survival after discharge | Relapse | Adverse events |
| --- | --- | --- | --- | --- | --- | --- | --- | --- |
| Kohn et al.  [1]  Prednisolone | 9 | >50,000/µL:  1, 2, 3, 3, 4, 5 and 9 days | >150,000/µL:  Tx_1_: 3, 5, 6, 6, 6, 11 and 13 days | ND | ND | ND | ND | ND |
| Putsche and Kohn  [2]  Prednisolone | 17 | ≥50,000/µL:  median 5 days (4-11 days) mean 6±2,2 days SD | ND | ND | ND | ##  ND* | ##  ND** | ND |
| Bianco et al.  [3]  Prednisone | 9 | #  > 40,000/µL:  median 7,5 days (3-12) mean 7.8± 3.9 days SD | ##  >160,000/µL:  median 13 days (5-32) | #  median 8 days (4-12) mean 8.3± 0.6 days SD^a^ | ND | ##  6-month:  78% | 6- month:  11% ^b^ | Grade 1 mild |

In studies with comparative analysis, (1) ## marked outcomes with no significant difference between drug protocols, (2) ### marked outcomes with significant difference between drug protocols and if the drug protocol was superior to the comparator, and (3) # marked outcomes with significant difference between drug protocols and if the drug protocol was inferior to the comparator.

Abbreviations: Aza, azathioprine; (Aza), adverse events described for azathioprine only; Cyclo, cyclosporine; Dex, dexamethasone; hIVIG, human intravenous immunoglobulin; (hIVIG) adverse events described for human intravenous immunoglobulin only; ND, outcome not specified for the protocol; ND*, outcome not specified for the protocol, but comparison of mortality described between prednisolone and a pooled group (prednisolone+vincinstine)+(prednisolone+azathioprine)+(prednisolone+cyclosporine); ND**, outcome not described for the protocol, but comparison of relapse described between prednisolone and a pooled group (prednisolone+vincinstine)+(prednisolone+azathioprine)+(prednisolone+cyclosporine); ref, reference range; SD, standard deviation; Vinc, Vincristine;

^a^ defined: initial presentation to discharge when clinical stable and platelet counts > 40,000/µL.

^b^ defined: a platelet count decrease of 50% compared to previous count or any count of <40,000/µL after initial response

^c^ defined: discharge when platelet counts ≥40,000/µL.

1. Kohn B, Engelbrecht R, Leibold W, Giger U. Clinical findings, diagnostics and treatment results in primary and secondary immune-mediated thrombocytopenia in the dog. Kleintierpraxis. 2000;45:893-+.

2. Putsche JC, Kohn B. Primary immune-mediated thrombocytopenia in 30 dogs (1997-2003). J Am Anim Hosp Assoc. 2008;44:250-7.

3. Bianco D, Armstrong PJ, Washabau RJ. A prospective, randomized, double-blinded, placebo-controlled study of human intravenous immunoglobulin for the acute management of presumptive primary immune-mediated thrombocytopenia in dogs. J Vet Intern Med. 2009;23:1071-8.
