# Supplementary material for: Immunomodulatory and immunosuppressive drug protocols in the treatment of canine primary immune thrombocytopenia, a scoping review
Source: Acta Vet Scand. 2021 Dec 27;63:54. doi: 10.1186/s13028-021-00620-z (PMC8721564; doi:10.1186/s13028-021-00620-z)
Supplement: Supplementary file 4 — Additional file 4: Outcomes from treatment with immunomodulatory or non-corticosteroid immunosuppressive drugs alone in canine primary ITP. [file 13028_2021_620_MOESM4_ESM.docx]

**Additional file 4.** Outcomes from treatment with immunomodulatory or non-corticosteroid immunosuppressive drugs alone in canine primary ITP.

| Study and protocol | Number of dogs | Platelet recovery time | | Duration of hospitalization | Survival to discharge | Survival after discharge | Relapse | Adverse events |
| --- | --- | --- | --- | --- | --- | --- | --- | --- |
| Yau and Bianco  [1]  Mycophenolate mofetil | 5 | >50,000/µL:  median 3 days (2-6) | ≥170,000/µL:  median 9 days (5-16) | median 3 days (2-7) | 100% | ND | ND | Grade 2 moderate |

Abbreviations: ND, outcome not specified for the protocol.

1. Yau VK, Bianco D. Treatment of five haemodynamically stable dogs with immune-mediated thrombocytopenia using mycophenolate mofetil as single agent. J Small Anim Pract. 2014;55:330-3.
