# Supplementary material for: Immunomodulatory and immunosuppressive drug protocols in the treatment of canine primary immune thrombocytopenia, a scoping review
Source: Acta Vet Scand. 2021 Dec 27;63:54. doi: 10.1186/s13028-021-00620-z (PMC8721564; doi:10.1186/s13028-021-00620-z)
Supplement: Supplementary file 5 — Additional file 5: Outcomes from treatment with adjunctive immunomodulatory or non-corticosteroid immunosuppressive drugs in canine primary ITP. [file 13028_2021_620_MOESM5_ESM.docx]

Additional file 5. Outcomes from treatment with adjunctive immunomodulatory or non-corticosteroid immunosuppressive drugs in canine primary ITP.

| Study and protocol | | Number of dogs | | Platelet recovery time | | Duration of hospitalization | | Survival to discharge | | Survival after discharge | | Relapse | | | Adverse events | |
| --- | --- | --- | --- | --- | --- | --- | --- | --- | --- | --- | --- | --- | --- | --- | --- | --- |
| Huang et al.  [1]  Prednisone/Dex + Aza | | 10 | | =40,000/µL:  median 6 days (4-12) | ref range:  median 15 days (12-21) | ND | | 100% | | ND | | ND | | | ND | |
| Kohn et al.  [2]  Prednisolone + Aza | | 3 | | >50,000/µL:  3.3 and 11 days | >150,000/µL:  6,10 and 22 days | ND | | ND | | ND | | ND | | | Grade 5 death (Aza) | |
| Putsche and Kohn  [3]  Prednisolone + Aza | | 3 | | ≥50,000/µL:  4.7 and 12 days | ND | ND | | ND | | ##  ND* | | ##  ND** | | | Grade 5 death (Aza) | |
| Huang et al.  [1]  Prednisone/Dex + Vinc | | 10 | | =40,000/µL:  median 4 days (2-10) | ref range:  median 10 days (3-42) | ND | | 90% | | ND | | ND | | | ND | |
| Balog et al.  [4]  Prednisone/Dex + Vinc | | 10 | | ##  ≥40,000/µL:  median 2.5 days (1-4) | ND | ##  median 4  days (3-5)^c^ | | ##  100% | | ##  6-month:  70%  1-year:  60% | | ND | | | Grade 1 mild | |
| Putsche and Kohn  [3]  Prednisolone + Vinc | | 6 | | ≥50,000/µL:  median 4 days (2-7) mean 4±2 SD | ND | ND | | ND | | ##  ND* | | ##  ND** | | | ND | |
| Huang et al.  [1]  Prednisone/Dex + hIVIG | | 6 | | = 40,000/µL:  median 5 days (2-10) | ref range:  median 12 days (2-13) | ND | | 83% | | ND | | ND | | | ND | |
| Bianco et al.  [5]  Prednisone + hIVIG | | 9 | | ###  >40,000/µL:  median 3.5 days (2-7) mean 3.7±1.3 SD | ##  >160,000/µL:  median 8 days (3-19) | ###  median 4 days (2-8) mean 4.2± 0.4 SD^a^ | | 100% | | ##  6-month: 100% | | 6-months: 11%^b^ | | | Grade 1 mild | |
| Balog et al.  [4]  Prednisone/Dex + hIVIG | | 10 | | ##  ≥40,000/µL:  median 2.5days (0-10) | ND | ##  median 5 days (1.5-10)^c^ | | ##  70% | | ##  6-month:  22%  1-year:  22% | | ND | | | Grade 1 mild | |
| Putsche and Kohn  [3]  Prednisolone + Cyclo | | 3 | | ≥50,000/µL:  5 and 9 days | ND | ND | | ND | | ##  ND* | | ##  ND** | | | ND | |
| Kohn et al.  [2]  Prednisolone + Aza + Vinc | 3 | | >50,000/µL:  2, 3 and 5 days | | >150,000/µL:  4, 5 and 13 days | | ND | | ND | | ND | | ND | ND | |  |

In studies with comparative analysis, (1) ## color marked outcomes with no significant difference between drug protocols, (2) ### color marked outcomes with significant difference between drug protocols and if the drug protocol was superior to the comparator, (3) # marked outcomes with significant difference between drug protocols and if the drug protocol was inferior to the comparator.

Abbreviations: Aza, azathioprine; (Aza), adverse events described for azathioprine only; Cyclo, cyclosporine; Dex, dexamethasone; hIVIG, human intravenous immunoglobulin; (hIVIG) adverse events described for human intravenous immunoglobulin only; ND, outcome not specified for the protocol; ND*, outcome not specified for the protocol, but comparison of mortality described between prednisolone and a pooled group (prednisolone+vincinstine)+(prednisolone+azathioprine)+(prednisolone+cyclosporine); ND**, outcome not described for the protocol, but comparison of relapse described between prednisolone and a pooled group (prednisolone+vincinstine)+(prednisolone+azathioprine)+(prednisolone+cyclosporine); ref, reference range; SD, standard deviation; Vinc, Vincristine;

^a^ defined: initial presentation to discharge when clinical stable and platelet counts > 40,000/µL.

^b^ defined: a platelet count decrease of 50% compared to previous count or any count of <40,000/µL after initial response

^c^ defined: discharge when platelet counts ≥40,000/µL.

1. Huang AA, Moore GE, Scott-Moncrieff JC. Idiopathic immune-mediated thrombocytopenia and recent vaccination in dogs. J Vet Intern Med. 2012;26:142-8.

2. Kohn B, Engelbrecht R, Leibold W, Giger U. Clinical findings, diagnostics and treatment results in primary and secondary immune-mediated thrombocytopenia in the dog. Kleintierpraxis. 2000;45:893-+.

3. Putsche JC, Kohn B. Primary immune-mediated thrombocytopenia in 30 dogs (1997-2003). J Am Anim Hosp Assoc. 2008;44:250-7.

4. Balog K, Huang AA, Sum SO, Moore GE, Thompson C, et al. A Prospective Randomized Clinical Trial of Vincristine versus Human Intravenous Immunoglobulin for Acute Adjunctive Management of Presumptive Primary Immune-Mediated Thrombocytopenia in Dogs. J Vet Intern Med. 2013;27:536-41.

5. Bianco D, Armstrong PJ, Washabau RJ. A prospective, randomized, double-blinded, placebo-controlled study of human intravenous immunoglobulin for the acute management of presumptive primary immune-mediated thrombocytopenia in dogs. J Vet Intern Med. 2009;23:1071-8.
